# Efficacy of Nicorandil in Preventing Myocardial Injury and Cardiovascular Outcomes in Patients Undergoing Percutaneous Coronary Intervention (PCI): A Systematic Review and Meta-Analysis

**DOI:** 10.7759/cureus.66938

**Published:** 2024-08-15

**Authors:** Hajra Tariq, Sara Ahmed, Sheraz Ahmed, Najma Hanif, Erum Anwar, Amrita Kumari, Calvin R Wei, Danish Allahwala

**Affiliations:** 1 Cardiology, Abbottabad International Medical College, Abbottabad, PAK; 2 Emergency Medicine, National Institute of Cardiovascular Diseases, Karachi, PAK; 3 Medicine, Islamic International Medical College, Islamabad, PAK; 4 Medicine, Sindh Medical College, Karachi, PAK; 5 Medicine, Sir Syed College of Medical Sciences for Girls, Karachi, PAK; 6 Medicine, Ziauddin Medical College and Hospital, Karachi, PAK; 7 Research and Development, Shing Huei Group, Taipei, TWN; 8 Nephrology, Fatima Memorial Hospital, Karachi, PAK

**Keywords:** systematic review and meta-analysis, major adverse cardiovascular event, primary percutaneous intervention, nicorandil, myocardial injury

## Abstract

Percutaneous coronary intervention (PCI) is a common procedure for treating coronary artery disease, but it carries a risk of periprocedural myocardial injury (PMI). This meta-analysis evaluated the efficacy of nicorandil, a hybrid compound with nitrate-like and potassium channel-opening properties, in preventing PMI during PCI. A comprehensive literature search identified 14 studies involving 1,762 patients, with 882 receiving nicorandil and 880 in the control group. The analysis revealed that nicorandil significantly reduced the incidence of PMI (RR: 0.73, 95% CI: 0.61-0.86) and major adverse cardiovascular events (MACE) (RR: 0.76, 95% CI: 0.58-0.99) compared to the control group. Nicorandil's cardioprotective effects are attributed to its ability to improve coronary blood flow, precondition the myocardium, and reduce oxidative stress and inflammation. These findings suggest that nicorandil could be a valuable adjunctive therapy during PCI, potentially improving patient outcomes. However, the study had limitations, including variations in drug administration methods and a lack of individual-level data for subgroup analysis. Future research should focus on optimizing dosing regimens and administration timing and comparing nicorandil's effectiveness with other cardioprotective agents.

## Introduction and background

Percutaneous coronary intervention (PCI) is a widely performed procedure aimed at restoring blood flow in patients with coronary artery disease (CAD) [1]. Despite its benefits, PCI is associated with the risk of periprocedural myocardial injury (PMI), a complication characterized by the release of cardiac biomarkers like troponin and creatine kinase-MB due to myocardial cell damage [2]. PMI is a significant clinical concern as it is associated with adverse outcomes, including an increased risk of future cardiovascular events and mortality [3]. Myocardial injury during PCI can result from various mechanisms such as distal embolization of atheromatous debris, side branch occlusion, coronary dissection, and microvascular dysfunction. The need for effective strategies to minimize PMI has led to the investigation of pharmacological interventions that can protect the myocardium during PCI [2]. 

Nicorandil, a hybrid compound possessing both nitrate-like and potassium channel-opening properties, has emerged as a potential therapeutic agent in this context [4]. It exerts its cardioprotective effects through several mechanisms. The nitrate component induces vasodilation, thereby improving coronary blood flow, while the potassium channel opener component preconditions the myocardium, rendering it more resistant to ischemic insults [5]. Additionally, nicorandil has anti-inflammatory and anti-apoptotic properties, further contributing to its potential benefit in reducing PMI [6]. These multifaceted actions of nicorandil make it a promising candidate for myocardial protection during PCI. The pharmacokinetics of nicorandil also contribute to its potential utility in the PCI setting. It has a rapid onset of action, with peak plasma concentrations achieved within 30-60 minutes of oral administration [7]. This characteristic allows for its timely administration before PCI procedures. Moreover, nicorandil has been shown to have a favorable safety profile, with minimal effects on blood pressure and heart rate, which is particularly important in the context of interventional procedures [6]. 

The efficacy and safety of nicorandil in the setting of PCI have been the subject of various clinical studies, with some suggesting a reduction in PMI and improved clinical outcomes, while others have yielded inconclusive results [8,9]. The variation in study outcomes may be attributed to differences in study design, patient populations, dosages, and administration protocols. Some studies have explored different dosing regimens, including pretreatment with oral nicorandil prior to PCI, intracoronary administration during the procedure, or a combination of both approaches. The optimal timing and route of administration remain subjects of ongoing research. 

Given the mixed evidence and the clinical importance of reducing PMI, a comprehensive evaluation through a meta-analysis is warranted. The aim of this meta-analysis is to systematically assess the available evidence on the efficacy of nicorandil in preventing PMI in patients undergoing PCI. This analysis will consolidate data from multiple studies to provide a more robust estimate of the effects of nicorandil, thereby aiding clinicians in making informed decisions regarding its use in clinical practice. 

## Review

Methodology 

Literature Search

A comprehensive literature search was conducted to identify relevant studies evaluating the efficacy and safety of nicorandil for PMI in patients undergoing PCI. Databases searched to find relevant articles included PubMed, Embase, Cochrane Library, and Web of Science. The search covered publications from January 2011 to July 25, 2024. Keywords and Medical Subject Headings (MeSH) terms included "nicorandil," "percutaneous coronary intervention," "PCI," "myocardial injury," "periprocedural," and "myocardial infarction." Boolean operators (AND, OR) were used to combine terms appropriately. Additionally, references of identified studies and relevant review articles were manually searched to ensure comprehensive coverage. The search was performed by two authors independently, and any disagreement between them was resolved through discussion. 

Study Selection 

The selection of studies involved a systematic and thorough screening process. Initially, two independent reviewers screened the titles and abstracts of all retrieved studies to identify those that potentially met the inclusion criteria. Studies eligible for inclusion were randomized controlled trials (RCTs) and prospective observational studies evaluating the efficacy and safety of nicorandil in the context of PCI, specifically reporting on the incidence of PMI defined by elevated cardiac biomarkers such as troponin or creatine kinase-MB and incidence of major adverse cardiovascular events (MACE). Studies that did not involve PCI or did not report specific outcomes related to PMI and MACE were excluded. Additionally, animal studies, case reports, review articles, and abstracts without full-text availability were excluded from the review. Full-text articles of potentially eligible studies were then thoroughly reviewed to confirm their eligibility. Any discrepancies between the two reviewers during the selection process were resolved through discussion and, if necessary, by consulting a third reviewer to reach a consensus. 

Data Extraction 

Data extraction was performed independently by two reviewers using a standardized data extraction form to ensure consistency and accuracy. Extracted data included study characteristics such as author, publication year, study design, sample size, and setting. Patient characteristics were recorded, encompassing age, gender, comorbidities, and outcomes. Primary outcomes focused on the incidence of PMI, defined by the elevation of cardiac biomarkers like troponin and creatine kinase-MB. Secondary outcomes included MACE, such as myocardial infarction, repeat revascularization, stroke, and cardiovascular mortality, as well as safety outcomes, including adverse drug reactions. Any discrepancies in data extraction between the two reviewers were resolved through consensus or by consulting a third reviewer. 

Statistical Analysis Plan 

The meta-analysis was conducted using RevMan software version 5.4.1 (Review Manager, Cochrane Collaboration). Statistical heterogeneity among the included studies was assessed using the I² statistic and chi-squared test, with an I² value greater than 50% indicating substantial heterogeneity. Depending on the level of heterogeneity observed, either a fixed-effects model or a random-effects model was employed to pool the data. For dichotomous outcomes, such as the incidence of PMI and MACE, risk ratios (RRs) with 95% confidence intervals (CIs) were calculated. A p-value less than 0.05 was considered significant. 

Results

**Figure 1:**
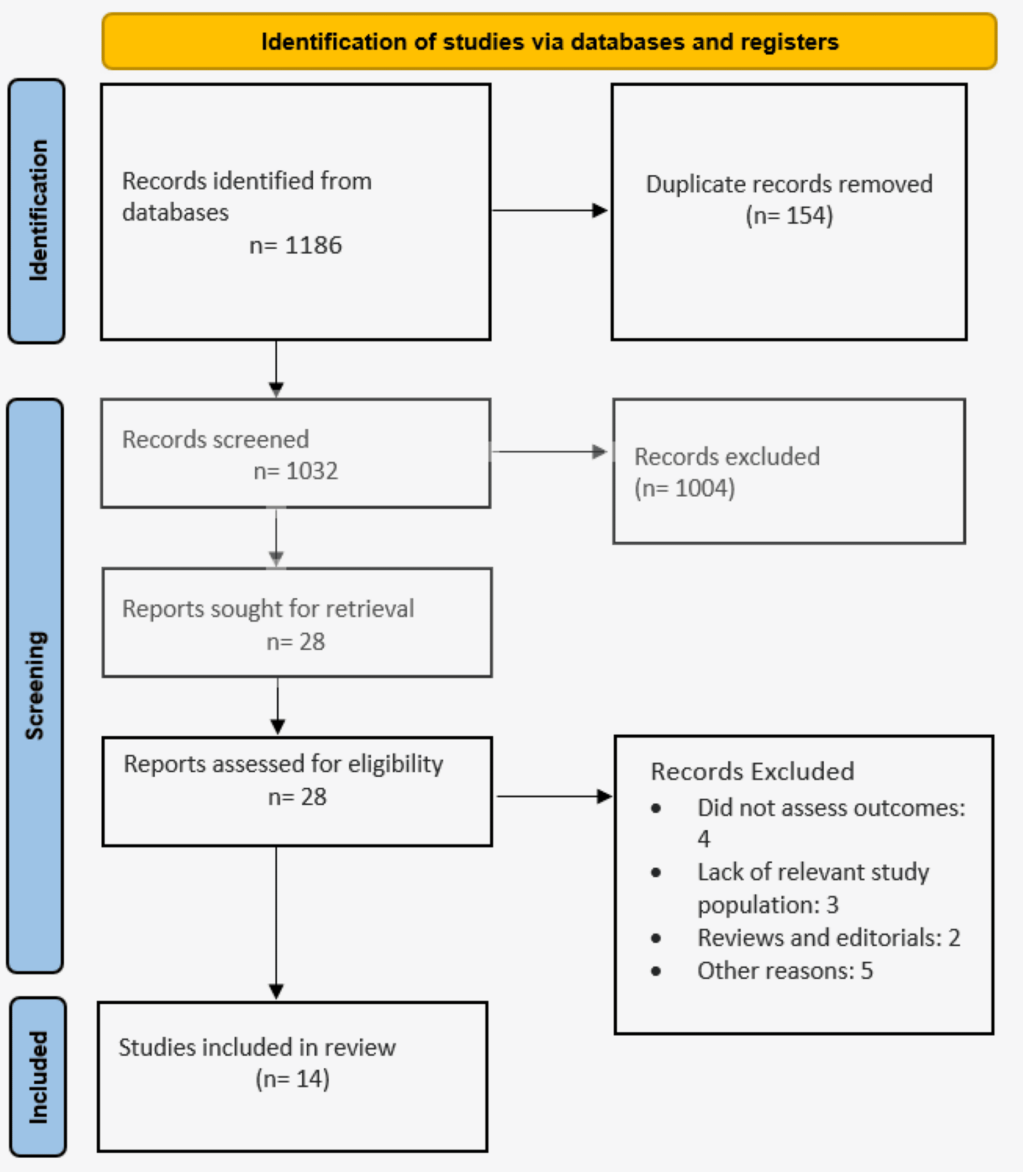
PRISMA flowchart (study selection process) PRISMA: Preferred Reporting Items for Systematic Reviews and Meta-Analyses

Following the Preferred Reporting Items for Systematic Reviews and Meta-Analyses (PRISMA) guidelines, we initially identified 1186 studies that met our inclusion criteria. The titles and abstracts of these studies were carefully reviewed to exclude those that were ineligible. Further screening of the full texts led to additional exclusions. Ultimately, 14 articles, were included in the analysis, with a total of 1762 patients (Figure 1). Of these, 882 patients (50.1%) received nicorandil, while 880 patients (49.9%) were in the control group. Table 1 presents the characteristics of the included studies.

**Table 1:** Characteristics of the included studies RCT: randomized controlled trial

Author	Year	Region	Design	Groups	Sample size	Age (years)	Males (n)
Ejiri et al. [10]	2018	Japan	RCT	Nicorandil	61	69.4	49
				Control	56	71.6	41
Feng et al. [11]	2019	China	RCT	Nicorandil	84	69.2	59
				Control	86	68.5	62
Hirohata et al. [12]	2014	Japan	RCT	Nicorandil	33	73	25
				Control	29	68	20
Hwang et al. [13]	2013	Korea	RCT	Nicorandil	41	66.2	20
				Control	40	65.3	25
Kawakita et al. [14]	2018	Japan	RCT	Nicorandil	94	74.3	69
				Control	95	75.4	69
Kim et al. [15]	2012	Korea	RCT	Nicorandil	54	61.7	32
				Control	55	60.4	27
Louis et al. [16]	2022	Egypt	Non-RCT	Nicorandil	40	56.3	32
				Control	40	54.4	35
Miyoshi et al. [17]	2017	Japan	RCT	Nicorandil	129	70	99
				Control	133	70.3	102
Nishimura et al. [18]	2014	Japan	RCT	Nicorandil	63	65	45
				Control	65	64	45
Shah et al. [19]	2024	Pakistan	Non-RCT	Nicorandil	45	62.4	38
				Control	45	63.1	30
Wang et al. [20]	2019	China	RCT	Nicorandil	60	58.2	35
				Control	57	56.7	41
Wang et al. [21]	2021	China	RCT	Nicorandil	59	54	47
				Control	60	55	47
Yang et al. [22]	2015	China	RCT	Nicorandil	45	57	31
				Control	47	53	31
Ye et al. [4]	2018	China	RCT	Nicorandil	74	54.7	56
				Control	72	56.3	54

Meta-Analysis of Outcomes 

**Figure 2:**
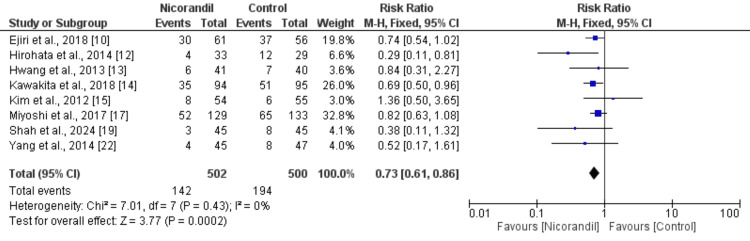
Comparison of PMI between two study groups PMI: periprocedural myocardial injury References: [10,12-15,17,19,22]

PMI: PMI was examined between two groups in eight studies. Specifically, PMI was reported in 142 out of 502 patients in the nicorandil group and in 194 out of 500 patients in the control group. As compared to the control group, the results indicated that nicorandil significantly reduced the incidence of PMI (RR: 0.73, 95% CI: 0.61-0.86) as shown in Figure 2. The study's results showed no heterogeneity (I^2^: 0%). 

**Figure 3:**
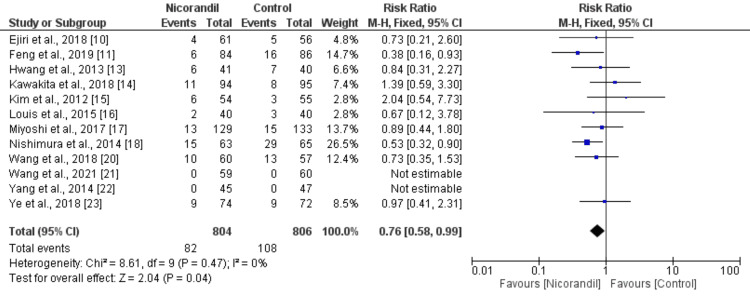
Comparison of MACE between two groups MACE: major adverse cardiovascular events References: [4,10,11,13-18,20-22]

MACE: MACE was analyzed in 12 studies comparing the two groups. Overall, MACE occurred in 190 out of 1610 patients, representing 11.8% of the total study population. Specifically, MACE was reported in 82 of 804 patients (10.2%) in the nicorandil group and in 108 of 806 patients (13.4%) in the control group. The findings demonstrated that nicorandil significantly reduced the incidence of MACE compared to the control group (RR: 0.76, 95% CI: 0.58-0.99) as shown in Figure 3. The analysis showed no heterogeneity among the studies (I²: 0%). 

Discussion 

Several significant findings emerged from this meta-analysis assessing the efficacy and safety of nicorandil for PMI in patients undergoing PCI. Firstly, there was a notable reduction in the frequency of periprocedural myocardial infarctions in the nicorandil group compared to the placebo group. Secondly, the risk of MACE was significantly lower in patients treated with nicorandil. A meta-analysis by Lu et al. demonstrated that early use of nicorandil in PCI patients significantly reduced PMI and MACE [23]. Li et al. observed that combining nicorandil with primary PCI decreased the incidence of cardiovascular events and no-reflow phenomenon (NRP) [24]. Research by Yi et al. found that nicorandil treatment during PCI reduced overall mortality, cardiovascular death, and heart failure in patients undergoing primary PCI (PPCI) and elective PCI (EPCI) [25]. Furthermore, this study indicated that there was no additional reduction in overall perioperative complications nor an increase in significant adverse cardiac events (MACE) in patients with angina pectoris undergoing PCI [25]. 

Wang et al. [21] found that the nicorandil group had lower peak levels of Mb, CK-MB, and cTnT-hs than the control group. This finding suggested that the nicorandil group had less myocardial necrosis, which was thought to be associated with the drug's myocardial protective effect. This could be one of the explanations for the patients' decreased risk of cardiac damage when using nicorandil. Second, nicorandil is known to decrease infarct size and encourage systolic function recovery following infarction, acting similarly to ischemic preconditioning [26,27]. Furthermore, nicorandil's ability to function as a potassium channel opener in addition to a nitrate may account for its ability to lower PMI and MACE. Myocardial resistance to ischemia damage is strengthened by its preconditioning qualities, and coronary blood flow is improved by its vasodilatory effects. Given that oxidative stress and inflammation are important components of the pathogenesis of myocardial damage, nicotinamide's capacity to decrease these two factors may also be responsible for some of its cardioprotective benefits [25,28]. 

The Impact of Nicorandil in Angina (IONA) research, a large-scale investigation into the myocardial protective effect of nicorandil in the high-risk group of patients with stable angina, revealed that, compared to the 15.5% of patients who received a placebo, 13.1% of patients who received oral nicorandil died from CAD or unexpectedly admitted for non-fatal myocardial infarction or chest pains. These results suggest that nicorandil may improve the prognosis of patients with unstable angina [29]. 

A comparative analysis of nicorandil with other cardioprotective agents used during PCI, such as adenosine or intravenous nitroglycerin, could provide a broader perspective on its position in clinical practice. Highlighting any advantages or limitations of nicorandil relative to these agents, in terms of effectiveness, side effects, and patient outcomes, could offer valuable insights. This comparison could help determine whether nicorandil presents superior benefits or fewer adverse effects, thus influencing its potential adoption as a preferred adjunctive therapy in PCI procedures. 

Emphasizing the need for long-term follow-up studies is crucial to assess the sustained benefits of nicorandil on survival rates and quality of life. Discussing data or trends observed in the studies regarding the long-term reduction in adverse cardiovascular events would provide valuable insights into the enduring impact of nicorandil therapy. Such long-term data would help determine whether the initial benefits observed with nicorandil are maintained over time, thereby solidifying its role in improving patient outcomes following PCI. 

There are certain limitations of the current meta-analysis. First off, not all drug administration methods are the same. There is a chance that certain papers will follow different procedures, such as oral, intravenous, or coronary artery administration, which could result in variations in methodology. Second, a lack of individual-level data prevented us from doing subgroup analysis based on characteristics including gender, comorbidities, and age. 

This research has significant clinical and research implications. Clinically, the findings suggest that nicorandil can be a valuable adjunctive therapy during PCI, reducing PMI and MACE, thereby improving patient outcomes. It highlights the need for incorporating nicorandil into standard PCI protocols. For research, the study underscores the importance of long-term follow-up to evaluate the sustained benefits of nicorandil, including its impact on survival rates and quality of life. Future studies should explore optimal dosing, administration timing, and comparative effectiveness with other cardioprotective agents to further refine its clinical application. 

## Conclusions

This meta-analysis demonstrates the efficacy of nicorandil in reducing PMI and MACE in patients undergoing PCI. Nicorandil's cardioprotective effects are attributed to its unique dual action as a nitrate and potassium channel opener, improving coronary blood flow and preconditioning the myocardium. The findings suggest that nicorandil could be a valuable adjunctive therapy during PCI, potentially improving patient outcomes. However, limitations such as variations in administration methods and a lack of individual-level data for subgroup analysis highlight the need for further research. Future studies should focus on optimizing dosing regimens and administration timing and comparing nicorandil's effectiveness with other cardioprotective agents to refine its clinical application.
